# Dissolution of Inorganic Lead (Pb) in Synthetic Sweat: Implications for Dermal Exposure and Occupational Risk

**DOI:** 10.3390/toxics14030258

**Published:** 2026-03-16

**Authors:** Efosa Obariase, John F. Reichard

**Affiliations:** Department of Environmental Health and Public Health Sciences, University of Cincinnati, Cincinnati, OH 45267, USA; dephs@uc.edu

**Keywords:** Pb nitrate, dissolution cell, bioaccessibility, ICP MS, dermal

## Abstract

Inorganic lead (Pb) poses a significant public health concern due to its toxicity and widespread industrial use. Dermal contact, an under-researched pathway of Pb exposure relative to inhalation and ingestion, is typically not factored into regulatory exposure limits because of the paucity of validated studies. This study investigated the influence of sweat on the bioaccessibility of inorganic lead for dermal absorption. Dissolution testing was conducted to determine the dissolution kinetics of inorganic Pb (lead nitrate) in synthetic sweat relative to deionized water (DIW). Particle sizes of samples ranged from 0.70 µm to 118 µm. Non-linear dissolution kinetics were observed in both DIW (control) and sweat. The iPb ion concentration in DIW after 3 h (test period) accounted for 100% of the initial mass of iPb, compared to 67% of the initial mass of iPb in sweat. Higher variability was observed in sweat (SD: 1.47 to 8.2) compared to DIW (SD: 0.80 to 3.88). Precipitation was observed in sweat but not in DIW. Wilcoxon rank-sum test indicated a statistically significant difference in dissolution between sweat and deionized water (Z = −4.50, *p* < 0.0001). Findings suggest that sweat composition limits the extent of dissolution of soluble inorganic Pb, thereby influencing its dermal bioaccessibility.

## 1. Introduction

Heavy metals are naturally occurring elements characterized by high atomic weight and density, and they tend to persist in the environment. They pose significant risks to human health because they can accumulate in living organisms. Elements such as arsenic, cadmium, chromium, and lead are extensively utilized across industrial sectors, including mining, battery manufacturing, smelting, pigment production, and electronics, presenting significant occupational and environmental exposure. Inorganic lead (iPb) is one of the most comprehensively studied hazardous heavy metals because of its widespread industrial application and well-established toxicological profile. The International Agency for Research on Cancer [1] classifies Pb as a probable human carcinogen, and chronic exposure has been associated with adverse health outcomes, including neurological impairment, renal dysfunction, hematological disturbances, and cardiovascular diseases [2,3]. Human exposure to Pb occurs primarily through inhalation of airborne Pb particles or fumes and ingestion of contaminated dust, food, or water. Dermal contact is an underexplored pathway of exposure to iPb, and this is particularly likely when sweat or other liquids found on the skin surface serve as a medium for dissolution.

Dermal exposure to Pb has gradually become an occupational health concern. With about 1.5 million workers in the United States annually exposed to inorganic Pb compounds in the workplace [4], no pathway to exposure should be disregarded. Industries frequently associated with Pb exposure include battery manufacturing and recycling, mining and smelting, construction and renovation, and ceramic, plastic, and pigment production. The dissolution behavior of iPb compounds on the skin surface is a key factor in the dermal exposure pathway. When Pb-containing dust contacts exposed skin in Pb-related industrial environments, absorption depends on the rates of these compounds (salts and oxides) that go into the solution with sweat and skin surface moisture, thereby releasing soluble iPb ions. In Pb-acid battery manufacturing, maximum skin loads as high as 80 µg/cm^2^ have been observed, attributed to Pb dust settling on the skin, direct handling of Pb compounds, and cross-contamination from personal protective equipment (PPE) and contaminated surfaces [5]. These iPb ions, and to some extent Pb salts, have some capacity to penetrate the stratum corneum (SC) of the epidermis and move past the deeper layers of the skin into the bloodstream, where they exert toxic systemic effects [6,7]. Therefore, the dissolution characteristics of iPb play a key role in determining its bioaccessibility and bioavailability. The bioavailability of a compound is the fraction of the applied dose that is absorbed into the body and reaches systemic circulation [8]. Bioavailability depends on bioaccessibility, which is the fraction of the toxicant released from its matrix (e.g., human sweat) that is available for absorption [8,9]. Key factors that influence the dissolution of iPb compounds include pH, sweat composition (ions, proteins, complexing agents, and dissolved organic carbon), temperature, and particle size [10,11,12,13].

Extensive research has been dedicated to elucidating the dissolution kinetics of powders, especially in the pharmaceutical and food industries [14]. Dissolution studies have attempted to determine key parameters, such as dissolution rate, for a variety of heavy metal salts or compounds (e.g., nickel, cobalt, Pb, and beryllium) [15,16,17,18]. The design of a dissolution assay must rigorously account for the analytical method’s accuracy and precision in quantifying the target analyte, and important experimental variables should be systematically evaluated. Such variables include test duration, sampling frequency, dissolution matrix (e.g., synthetic sweat solution), system temperature, the material composition of the containment vessel, and agitation. Furthermore, comprehensive characterization of the compound’s physicochemical properties is essential, as these attributes critically inform the selection and optimization of the test system.

Artificial sweat formulations have been employed as proxies for human sweat in several studies investigating the dissolution kinetics of various metals. The primary advantage of using synthetic sweat compared to actual human sweat lies in the ability to control experimental variability, thereby enhancing reproducibility and facilitating cross-study comparisons [19]. Several artificial sweat formulations have been described in the literature; however, no formulation fully replicates the complex composition of authentic human sweat. Some recipes aim to replicate the full complexity of human sweat [20], whereas others use far simpler mixes—such as the eight-component ISO artificial sweat for textile color-fastness testing [19], or the three-ingredient solution specified in the European Standard EN 1811 for nickel release assays [19,21,22,23]. In a comprehensive review of forty-five sweat formulations, Stefaniak & Harvey documented a broad range of compositions, with sodium, chloride, lactic acid, and urea emerging as the most common constituents [24]. Variations in the formulation and pH of artificial sweat can profoundly influence the solubility of metals. Multiple studies show that more acidic formulations of sweat (e.g., NIHS 96-10) [25] promote greater metal bioaccessibility compared to neutral sweat [10,26,27]. Sweat models that are not designed to meet specific quality standards tend to be more complex, reflecting the full spectrum of human sweat constituents [19]. Investigating inorganic Pb dissolution requires a matrix that contains components of human sweat that have the greatest potential to react with Pb^2+^ ions to form precipitates and thus reduce its solubility. Pb^2+^ forms poorly soluble precipitates with a range of sweat-relevant anions like chloride (Cl^−^), bromide (Br^−^), iodide (I^−^), sulfate (SO_4_^2−^), hydroxide (OH^−^), carbonate (CO_3_^2−^), phosphate (PO_4_^3−^), and fluoride (F^−^) [28,29,30,31,32]. Since all these anions are present in human sweat S [33], excluding any of these anions could compromise the accuracy of in vitro skin dissolution models [24]. Moreover, amino acids, which are common constituents of sweat, can both enhance and inhibit metal solubility [16].

This study tests the hypothesis that interactions between the components of sweat and Pb^2+^ reduce the dissolution rate of iPb compared to its dissolution in water due to the formation of poorly soluble salt complexes. A complex artificial sweat formulation was employed to simulate human sweat and quantify the dissolution of inorganic Pb through the measurement of iPb ion concentration. Lead nitrate was selected as the Pb^2+^ source because of its high solubility compared to other inorganic Pb salts (aside from lead acetate). The sweat formulation was adopted from a study conducted by Harvey et al. [20], which made use of an aqueous solution of sixty-one constituents at concentrations that mimic human sweat. Sebum was excluded because prior work showed it exerted a negligible effect on metal release, whereas the sweat fraction of skin surface film liquids (SSFL) is the primary driver of dissolution [34].

## 2. Materials and Methods

### 2.1. Materials

All chemicals used were reagent-grade. Mixed cellulose ester (MCE) membrane filters with 47 mm diameter (Sigma-Aldrich Inc., St. Louis, MO, USA) and a pore size of 0.2 µm were used because this pore size can filter out suspended solids while allowing dissolved ions (Pb^2+^) to pass through [35]. Their uncharged membranes have a low affinity for metal cations, reducing the risk of Pb absorption into the filter material. Their hydrophilicity ensures efficient filtration without the need for pre-wetting. They are compatible with water and dilute acids. Nylon or polytetrafluoroethylene membranes were not used because they are anionic and have high binding affinities for charged metal ions, or they require pre-wetting with organic solvents, which could interfere with Pb measurements [35].

### 2.2. Methods

An experimental approach was adopted in this study involving two steps. First, the study powders were characterized to determine particle size and morphology. Next, dissolution assays were conducted to evaluate the bioaccessibility of inorganic Pb powders.

#### 2.2.1. Study Powders

Particle size distribution and morphology of bulk lead nitrate powders (lead (II) nitrate, CASRN 10099-74-8, Sigma-Aldrich Inc., St. Louis, MO, USA) were analyzed using scanning electron microscopy (SEM). Lead nitrate particles were imaged at 200× and 50× magnifications, with 20 images (40 in total) captured at each magnification. Prior to imaging, samples of Pb nitrate granules were sputter-coated with a thin layer of gold–palladium using a Denton Desk V coater operated at 15 mA for 5 s to enhance conductivity. Imaging was conducted on a Thermo Scientific Apreo +C SEM at an accelerating voltage of 5.0 kV and a beam current of 0.1 nA to optimize resolution and surface detail. SEM images (TIF format) were analyzed using Fiji Image J software (Image J version 1.54p with the Fiji distribution, National Institutes of Health, Bethesda, MD, USA). For analysis, raw SEM micrographs originally stored on a flash drive were first imported into Fiji Image J software and converted to 8-bit grayscale. Each image was calibrated to the scale bar included in the SEM image to ensure accurate dimensional measurements. Prior to the measurement of Pb nitrate granules, images were pre-processed by applying background subtraction and contrast enhancement to improve particle boundary detection. Thresholding was then used to binarize the images, isolating Pb nitrate particles from the background. The “Analyze Particles” tool in Fiji Image J was used to quantify particle size, where settings were optimized to exclude background noise and partial edge particles. Measurements were extracted as equivalent circular diameter (ECD), area, and perimeter for each particle. Data from replicate micrographs were combined to generate particle size distributions, which were used to characterize the morphology of the Pb nitrate particles for subsequent statistical analysis.

#### 2.2.2. Preparation of Synthetic Sweat Solution

Four liters of fully aerated 18 MΩ-cm DIW was added to a 6 L Erlenmeyer flask on a magnetic hotplate and warmed to 32.3 °C with continuous stirring using a magnetic stir bar. The respective masses of electrolytes and ionic constituents were added to the flask, followed by organic acids, carbohydrates, amino acids, nitrogenous substances, and vitamins; sodium chloride was added sequentially to match the median sodium level in human sweat (Table A1—Appendix A). This was done with the aid of an analytical balance (Mettler Toledo, Cincinnati, OH, USA). For sweat constituents with extremely low concentrations, diluted stock solutions were prepared with DIW. The pH of the formulated sweat solution was titrated to 5.3 through the addition of 1 M ammonium hydroxide while monitoring pH with a calibrated pH electrode (Accumet BASIC AB15 pH meter, Fisher Scientific, Pittsburgh, PA, USA). The final volume of solution was brought to 6 L with DIW, and the final pH was verified. The artificial sweat solution was filtered through a 0.2 µm pore size MCE membrane filter to minimize the opportunity for microbial growth and stored in the refrigerator at −4 °C.

#### 2.2.3. Dissolution Assay

**Figure 1:**
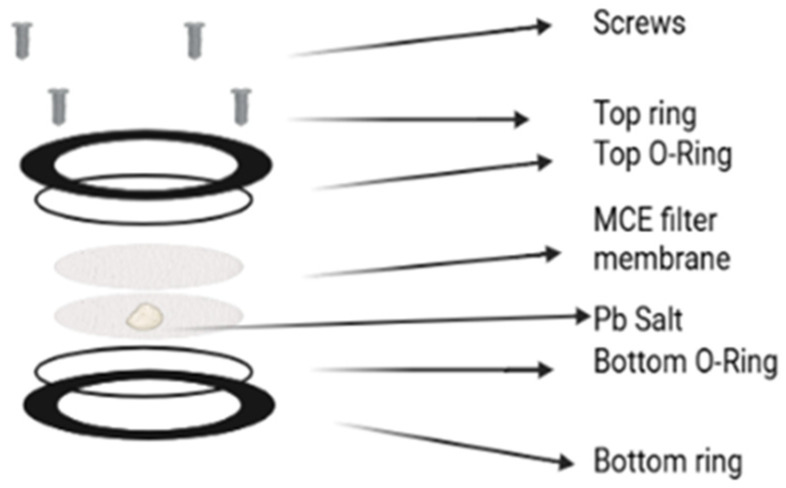
Dissolution cell assembly.

**Figure A1:**
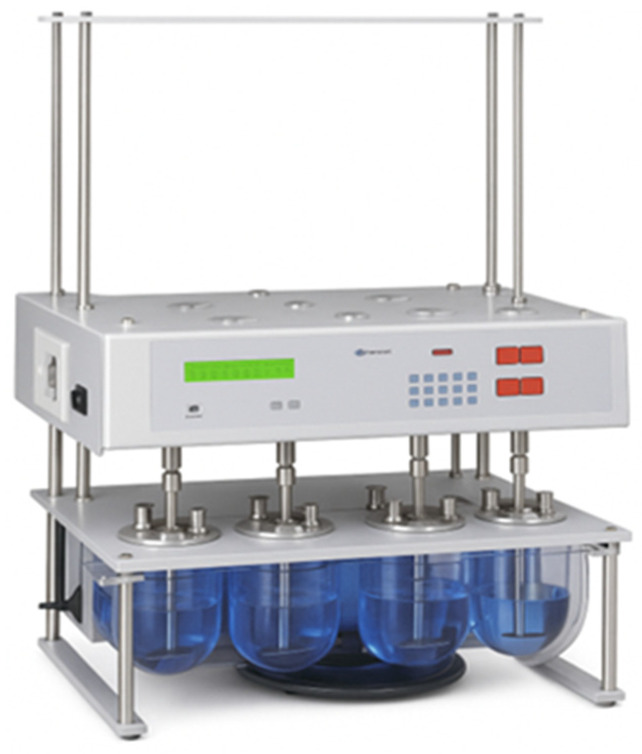
Varian VK 7000 dissolution apparatus.

The Varian VK 7000 dissolution apparatus (used in this study) consists of six 1 L dissolution vessels with a motorized paddle for each vessel to provide agitation (Figure A1: Appendix B). Each experimental condition was run in triplicate. A total of 400 mL of fully aerated DIW was measured into the first three vessels of the dissolution apparatus, labeled 1, 2, and 3, respectively, and 400 mL of the formulated sweat solution was measured into the last three dissolution vessels, labeled 4, 5, and 6, respectively. The Pb powder was prepared for dissolution analysis by measuring 125 mg of lead nitrate powder with a calibrated analytical balance onto pre-weighed MCE membrane filter papers (Sigma Aldrich Inc., St. Louis, MO, USA. Product number: GSWP04700). Powders were sealed between two membrane filters (17.35 cm^2^) in a plastic ring assembly called the “dissolution cell” (Figure 1). The above-stated mass value exceeds skin loading values cited in the literature, which range between 0.2 and 80 µg/cm^2^ [5,36]. Based on the initial Pb nitrate load and the surface area of the membrane filters (17.35 cm^2^) in the dissolution cell assembly, the maximum surface Pb nitrate load is computed as 125 mg/17.35 cm^2^, approx. 7204 µg/cm^2^. The final concentration of Pb nitrate in solutions, assuming full dissolution, is 0.3125 mg/mL, which is less than 1/1000th (~0.05%) of its solubility limit in water, 597 mg/mL at 25 °C [37].

The dissolution cell was gently lowered to the bottom of the vessel containing 400 mL of either DIW or synthetic sweat solution, ensuring a vertical distance of at least 25 mm between the dissolution cell and the rotating paddle. The dissolution apparatus was set to RUN with paddle rotation set at 50 rpm and water bath temperature set to 32.3 ± 0.5 °C. The assay was run for 180 min (3 h). One mL samples were taken from all six vessels at eight timepoints, 0 (baseline), 5, 15, 30, 60, 90, 120, and 180 min (*n =* 24) to quantify dissolution kinetics. Paddle rotation was “stopped” during sample collection, and samples were taken from the midpoint of the matrix solution, and all samples were collected within 20 s.

#### 2.2.4. Sample Preparation and ICP MS Analysis

At fixed timepoints during the dissolution process, 1 mL of dissolution sample was collected and diluted in DIW to 1:100 in 100 mL volumetric flasks. A total of 4 mL of each diluted sample was transferred to 15 mL conical tubes for analysis. Samples were acidified with 1 mL of 10% nitric acid (HNO_3_) to stabilize Pb ions in solution, bringing the final nitric acid concentration to 2% *v*/*v*. A total of 50 µL of 100 ppb Yttrium (89Y) internal standard (Sigma Aldrich, Pcode:102838014) was added to all samples to correct for instrument drift and physical interference. Samples were analyzed using Inductively Coupled Plasma Mass Spectroscopy (ICP MS) (Agilent 7500ce ICP MS, Agilent Technologies Inc., Santa Clara, CA, USA), following guidance set out in the EPA method 6020B within the SW-846 test methods manual [38]. ICP calibration standards (8 ppb, 40 ppb, 200 ppb, 1 ppm, and 5 ppm) were prepared using known Pb concentrations of analytical reagents (1 g/L lead standard for ICP, Sigma Aldrich), and the sample concentration was determined with reference to these standards.

### 2.3. Data Analysis

#### 2.3.1. Particle Size Data

Data obtained on the particle surface area of lead nitrate particles following analysis of SEM images with Fiji Image J version 1.54p was used to compute the particle size diameter in µm using the formula in Equation (1) below (using Microsoft Excel^®^):

(1) $$\mathrm{Particle}\;\mathrm{size}\;\mathrm{diameter}\;(2r)\;=2*\sqrt[{2}]{\frac{A}{\pi }}*1000$$

where *A* represents the surface area of the particle, and π is a constant (~3.1416). This data was subsequently used to generate particle size distribution with the aid of JMP Software, Student Edition 19.0.1 (SAS Institute Inc., Cary, NC, USA).

#### 2.3.2. Dissolution Assay Data

Summary statistics were calculated, including mean and standard deviation for Pb^2+^ concentration measurements with replicate samples using Microsoft Excel. Normality was tested using the Shapiro–Wilk tests. Non-parametric statistical analysis was performed using the Wilcoxon rank-sum test due to a right-skewed non-normal distribution of dissolution data. Statistical significance was set at α = 0.05. Dissolution curves were plotted from data obtained from DIW and synthetic sweat.

## 3. Results

### 3.1. Particle Size Analysis

**Figure 2:**
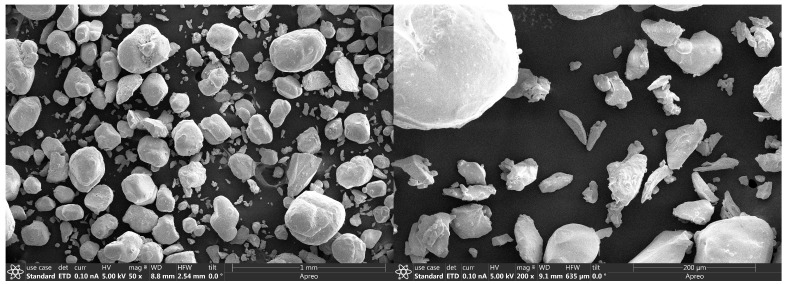
Micrographs produced by scanning electron microscopy of lead nitrate at different particle sizes: (**left**) Sample 1 at 50× magnification and (**right**) Sample 2 at 200× magnification. Scale bar = 1 mm.

**Figure 3:**
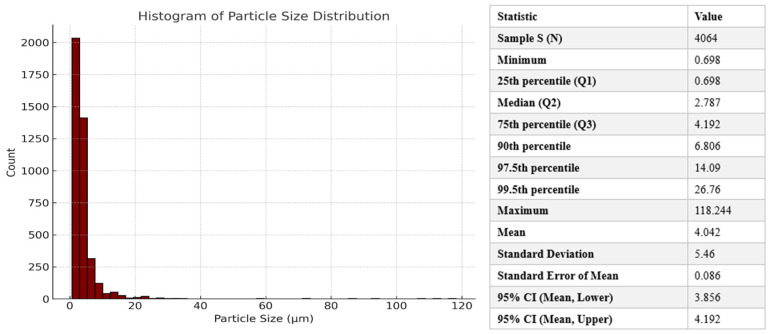
Particle size distribution for lead nitrate samples generated using JMP statistical software.

Particle size analysis of samples of lead nitrate powder (Figure 2) revealed particle size diameters within the range of 0.70 µm to 118 µm with mean and median values of 4.02 µm and 2.87 µm, respectively (Figure 3). Of the total number of observed particles, 99.5% were below 26.8 µm. The interquartile range was between 0.81 and 4.19 µm.

The membrane filters selected for use in the dissolution assays have a pore size of 0.22 µm, and Pb nitrate particles that are much larger are not able to pass through this membrane into the surrounding medium (sweat or deionized water) unless they undergo significant size reduction, and even then, the diffusion rate of suspended particles will be substantially less than the diffusion of the dissociated ions (Pb^2+^ and NO_3_^−^) in the solution. This assumption was incorporated into the study design to assess in vitro bioaccessibility. Particle size is a critical factor in dissolution, with smaller particle sizes presenting a larger surface area relative to mass, so they go into solution at a faster rate than the larger particles [39,40]. Particle size and distribution also determine the mean hydrodynamic boundary layer generated on the surface of particles undergoing dissolution [41], consequently influencing the rate of diffusion.

### 3.2. Dissolution Assay

**Figure 4:**
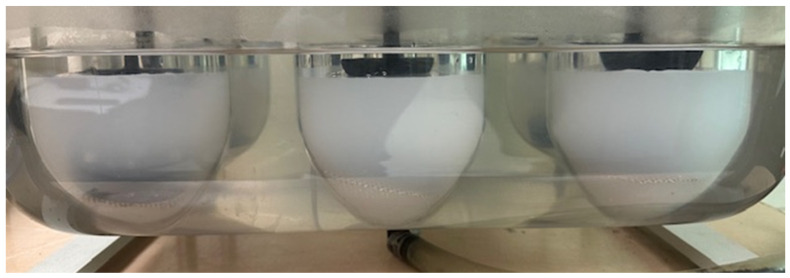
Cloudy solution forms 5 min after commencing dissolution in artificial sweat.

A cloudy solution was observed approximately five minutes into the dissolution experiment (Figure 4), indicating the onset of precipitation due to the interaction of Pb ions with anions in the synthetic sweat formulation, such as (PO_4_^3−^, SO_4_^2−^, OH^−^, and CO_3_^2−^), which form sparingly soluble salts.

**Figure 5:**
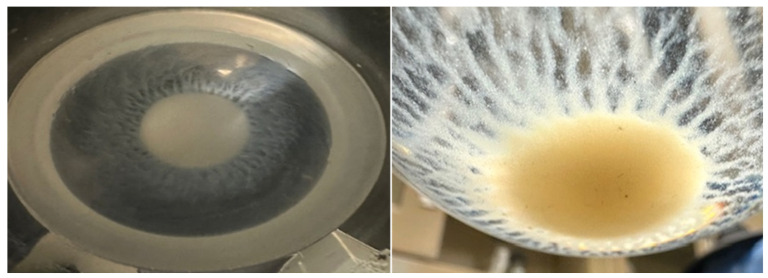
Representative precipitate formation at the bottom of the dissolution vessel in synthetic sweat, no precipitate is observed in deionized water. 1. Top view looking down towards the bottom of the dissolution vessel (**left**). 2. Same sample looking from the side (**right**).

In the presence of ongoing agitation, the cloudiness eventually cleared after about 30 min with the appearance of a precipitate at the bottom of the vessel, suggesting growth or aggregation of smaller suspended particles into larger particles that were too massive to remain suspended (Figure 5).

**Figure 6:**
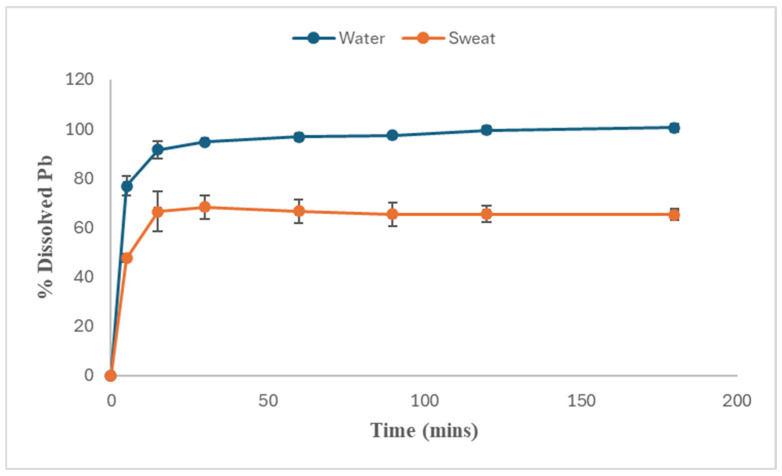
Plot of dissolved Pb (lead nitrate) over time in deionized water and artificial sweat. Error bars represent standard deviation for all points.

The dissolution of Pb nitrate in DIW described a non-linear curve, with 100 percent dissolution of the initial mass of Pb nitrate achieved within 120 to 180 min of the start of the assay. The dissolution curve shows a rapid increase in percent of dissolved Pb ions, reaching approximately 92% of the initial mass within the first 15 min (Figure 6), indicating a fast initial dissolution phase. On the linear scale, the dissolution rate reduces significantly after this rapid phase, and the curve plateaus as most of the initial mass of Pb has gone into solution. The rapid initial phase (Figure 6) is due to the large concentration gradient between solute and solvent, promoting diffusion of Pb^2+^ and NO_3_^−^ ions into the solution:

Pb(NO_3_)_2_ (s) ↔ Pb^2+^ (aq) + NO_3_^−^ (aq) (2)

As the initial mass of Pb goes fully into solution, the rate of dissolution slows down and dissolution stops. The standard deviation decreased from 3.88 after 5 min to 0.82 after 180 min, indicating improved consistency among replicates as dissolution progressed.

The dissolution profile of Pb nitrate in synthetic sweat (pH 5.3) showed a biphasic release pattern (Figure 6), with rapid dissolution occurring during the first 15 min of 66.56% ± 8.16 of the initial mass (which is approx. 32.98% less Pb^2+^ ion concentration than in DIW) and reached a maximum dissolution of 68.26% ± 4.76 after 30 min. After the initial dissolution phase, there was a gradual decline in Pb ion concentration between 30 and 90 min, after which an equilibrium state was attained. At the 180 min timepoint, only 65.33% ± 2.48% remained dissolved, which is 34.67% less than in DIW. Figure 6 compares the plots of percentage Pb dissolved over time in deionized water to artificial sweat. There is a similarity in the initial rate for DIW and sweat in the first 5 min, which slows over time over 15 min. At 180 min, the equilibrium between Pb ions in solution and those not in solution (complexed) is approx. 30% lower in sweat than in water. The slight decline in the dissolution curve observed after the maximum dissolution in synthetic sweat was not observed in the curve generated by deionized water.

### 3.3. Statistical Analysis

Data obtained from the DIW assay did not pass the normality test (W = 0.58882, *p*-value = 0.0001176), with *p* < 0.05. Data from the synthetic sweat assay (W = 0.58739, *p*-value = 0.0001132) had a similar outcome.

## 4. Discussion

In vitro dissolution testing can be used as a rapid and inexpensive method of predicting in vivo absorption of chemicals by characterizing their solubility and dissolution [42]. Dissolution in sweat is a key factor in the dermal absorption of the iPb. The dissolution assay of Pb nitrate in artificial sweat is a model that enables the characterization of the dissolution kinetics of inorganic Pb (and other contaminants) for human dermal exposure estimates.

The dissolution mechanism of Pb nitrate is by diffusion, as is common in the dissolution of most solids in liquids [43]. The contrasting dissolution profiles between deionized water and artificial sweat demonstrate the profound influence of sweat composition on iPb bioaccessibility. In deionized water, Pb nitrate exhibited rapid dissolution, reaching approximately 92% dissolution after 15 min, and >99% at the conclusion of the experiment, which is consistent with its high solubility in pure aqueous systems [44]. However, artificial sweat showed markedly different behavior with lower maximum dissolution of approximately 68% of the initial amount after 15 min, and approximately 65% at the conclusion of the experiment, as well as significant precipitate formation that indicates complex ion interactions that limit bioavailable Pb concentrations. Jukubiak et al. [42] described precipitation in their Unified Dissolution–Precipitation Model as a complex process involving two major molecular events.

(1) Particle growth where discrete dissolved molecules aggregate onto an already existing particle, which is defined as:
  (3) $$Particle\;growth\;rate=k_{growth}*A_{s}*C$$
  where *As* is the amount of solid material, *C* is the concentration of material, and kgrowth is the reaction rate of the solid particles with dissolved molecules.
(2) Particle nucleation involves the spontaneous generation of aggregates out of a dissolved compound from two or more molecules of solute (or very small aggregates) and is expressed in kinetic terms in Equation (4) below:
  (4) $$Nucleation\;rate=k_{nuc}*C^{\alpha }*V$$
  where *k_nuc_* is the nucleation rate constant, *α* is the molecularity index, representing the average number of molecules involved, and *V* is the compartment volume.

The observed precipitation in artificial sweat solutions suggests the formation of insoluble Pb complexes with sweat constituents not present in DIW. The observed initial cloudiness after 5 min, followed by partial “settling” with the formation of precipitates at the bottom of the vessel after 30 min, indicates a dynamic equilibrium between dissolution and precipitate formation. Precipitate formation could be attributed to Pb interactions with chloride, phosphate, bromide, iodide, sulfates, carbonates, hydroxides, lactate, and other anionic species present in this model of human sweat [45,46,47,48]. The maximum concentration of Pb nitrate achievable in both matrix solutions (0.03125 g/100 mL) was less than 1% of its aqueous solubility of 59.7 g/100 mL (at 25 °C). This ensured sink conditions, maintaining a concentration gradient between the surface of the dissolving compound and the bulk dissolution medium, which provides the thermodynamic driving force for dissolution [49]. Hence, the precipitate formation in the synthetic sweat formulation can only be explained by complexation following the interaction of Pb ions with anions in solution. At maximum dissolution, the sweat solution is supersaturated, the solute has reached its maximum solubility, and equilibrium has been exceeded. Hence, the formation of precipitates reestablishes equilibrium (Q > *Ksp*). To get a better understanding of the process and determine what salts precipitated out of solution, we define two concepts.

(1) **Reaction Quotient (Q)** measures the relative amounts of products and reactants present in a chemical reaction at a specific point in time [50] and is defined as:
  (5) $$Q=\frac{{\left[products\right]}^{coefficients}}{{\left[reactants\right]}^{coefficients}}$$
  For instance, when an ionic salt like PbSO_4_ dissolves and is represented by the chemical equation below:
  (6) $$PbSO_{4(s)}⇌{\;{Pb}^{2+}\left(aq\right)+SO}_{4}^{2-}\;\left(aq\right)$$
  **Reaction Quotient (Q)** $=\left[{Pb}^{2+}\right][{SO}_{4}^{2-}]/PbSO_{4}$.
  Q only considers concentration in solution, so the denominator = 1, because PbSO_4_ is solid and not in solution.
(2) **Solubility product constant** (***Ksp***) is an equilibrium constant that quantifies the solubility of sparingly soluble ionic compounds in water. *Ksp* is an indication of the aqueous solubility of a solid and is normally determined at 298 K (25 °C). The higher the *Ksp* value, the more soluble the compound is. The relationship between Q and *Ksp* is such that the solution is unsaturated when Q < *Ksp*, thus allowing more ions. When Q = *Ksp*, the solution is saturated and said to be in equilibrium. And when Q > *Ksp*, you have a supersaturated solution, at which point precipitation typically occurs [51]. This is the situation in Figure 6, where a decline in percent of dissolved Pb ions is apparent after the 30 min timepoint.

Continuing with the example of PbSO_4_:

(7) $$Ksp=\left[{Pb}^{2+}\right][{SO}_{4}^{2-}]\;\;(\mathrm{at}\;\mathrm{equilibrium})$$

From *Ksp* values, the critical [Pb^2+^] concentration or its molar solubility can be determined. This is the equilibrium concentration of Pb ions in solution, which, once exceeded, results in precipitate formation. It is the concentration of Pb ions when Q = *Ksp*. For instance, critical [Pb^2+^] for PbSO_4_ in the formulated sweat is computed below using Equation (7):

*Ksp* for PbSO_4_ = 1.6 × 10^−8^

$$Ksp=\left[{Pb}^{2+}\right][{SO}_{4}^{2-}]$$

1.6 × 10^−8^ = [*X*] [*X*]

1.6 × 10^−8^ = *X*^2^

Critical [Pb^2+^] = [X] = SQRT (1.6 × 10^−8^) = 1.26 × 10^−4^ M

When Pb nitrate goes into solution, releasing its components Pb^2+^ and NO_3_^−^, there is a high probability that Pb^2+^ could combine with other anions in sweat to form complexes. NO_3_^−^ ion, on the other hand, is a weak conjugate base, and rather than accept a proton in sweat or water, it remains in solution as a spectator ion.

The first column in Table 1 below is a list of possible Pb complexes based on the ionic composition of the formulated sweat solution. Based on precipitate color, lead iodide and lead sulfide (highlighted in blue) were excluded because the precipitate formed in the sweat solution was whitish in color. Next, lead fluoride, lead chloride, and lead bromide (highlighted in green) were excluded based on their critical Pb ion concentrations because these were all higher than the maximum Pb ions that could be provided in solution (3.77 × 10^−4^ M), so they are very unlikely to form Pb complexes that would precipitate out of solution. 

Lead phosphate is most likely to form a precipitate because it has the lowest *Ksp*; phosphate ions are in abundance in the sweat solution, and its precipitation is also rapid and irreversible. This is supported by a dissolution study conducted by Todd Niemeier at NIOSH laboratories, where the precipitate formed was Pb phosphate. Lead hydroxide (Pb (OH)_2_) and lead carbonates (PbCO_3_) are next in line based on *Ksp* values. However, studies by Huang et al. confirm that these latter salts only precipitate at neutral-to-alkaline pH, where they have the lowest solubility [2]. Therefore, at a pH of 5.3 (used in this experiment), we expect to have Pb phosphate and Pb sulfate precipitate in the sweat formulation, as both have the characteristic white precipitate color and low *Ksp* values.

Precipitate formation helps the sweat solution restore equilibrium, as can be seen in Figure 6, where the curve equilibrates or plateaus before the 180 min mark. The slight decline in dissolved Pb after the maximum dissolution point can also be plausibly explained by the “Oswald ripening” phenomenon, wherein smaller particles dissolve in solution only to redeposit on larger particles and make them “grow” bigger [53,54], because it is thermodynamically favored.

**Figure 7:**
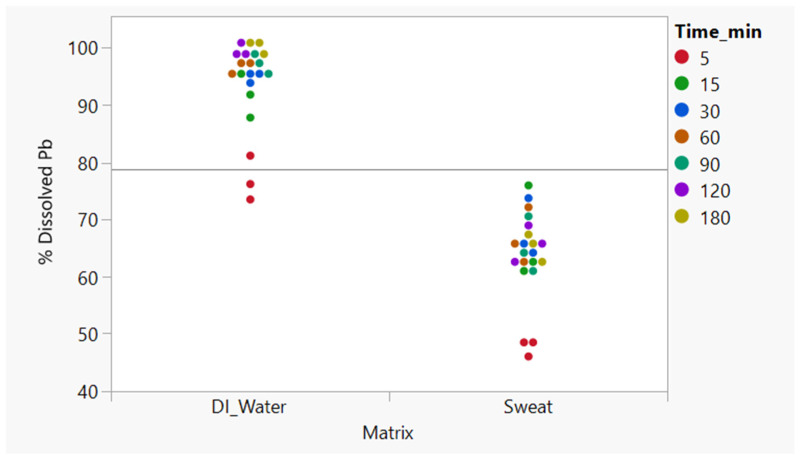
Non-parametric Wilcoxon rank-sum test on the Pb ion concentrations at all timepoints in deionized water vs. sweat matrices. Colors used to describe replicates at different timepoints (Z = −4.50, *p* < 0.0001).

The Wilcoxon rank-sum test (Figure 7) confirmed that the observed difference in dissolution rate between DIW and sweat was highly significant (Z = −4.50, *p* < 0.0001). This rules out the possibility that the observed differences are due to random variation. The significantly lower dissolution observed in the sweat matrix suggests that ionic and organic constituents of sweat limit the dissolution of Pb relative to the aqueous environment of deionized water and thus limit the bioaccessible Pb fraction. The null hypothesis that dissolution in sweat is the same in DIW and synthetic sweat (H_0_) is rejected in this case.

These findings emphasize the importance of measuring bioaccessible fraction rather than total lead content in occupational health risk-exposure assessments to avoid overestimation of bioavailable lead by not accounting for precipitation and complexation. The temporal dissolution patterns also suggest that prolonged skin contact of greater than 30 min might not proportionally increase lead uptake due to precipitation equilibria. This has implications for developing exposure limits, administrative controls, and protective equipment recommendations for Pb-exposed workers.

### Limitation

Although the study design reflects careful consideration, the in vitro experimental conditions may not fully replicate real-world exposure scenarios. Since the Pb nitrate powder is sandwiched between two membrane filter papers in the dissolution cell, the dissolving solution (sweat or deionized water) must first diffuse through the pores of the filter to reach the solid Pb particles, introducing an additional mass transfer resistance that may reduce the observed dissolution kinetics. As a result, the experimentally determined rate constant is likely somewhat lower than it would be if the Pb particles were not confined between porous filters. The extent of this resistance remains unknown. However, this is controlled since the same setup was used for both DIW and synthetic sweat. Furthermore, it is unclear how long it takes for the dissolving solution to displace the air initially present around the particles between the filters. This delay could mean that the Pb particles are not immediately exposed to the synthetic sweat upon immersion, potentially leading to lower measured dissolution rates compared to Pb particles in direct contact with a thin layer of sweat on the skin surface.

## 5. Conclusions

This study employed a comprehensive sweat formulation to demonstrate that sweat chemistry modifies the dissolution behavior and dermal bioaccessibility of soluble inorganic Pb (like Pb nitrate used in this study). The statistically significant difference between sweat and aqueous dissolution highlights the limitations of water-based models for estimating dermal lead exposure. These findings suggest that total Pb mass may overestimate dermal risk if sweat-mediated precipitation is not considered. Incorporating biologically relevant sweat chemistry into occupational exposure assessments will improve the accuracy of dermal risk models and support more evidence-based exposure control strategies.

## Figures and Tables

**Table 1:** Lead salts with their solubility product constant, *Ksp*, characteristic color, and critical Pb ion concentrations.

Pb Compound	Formula	*Ksp*	Precipitate Color	Critical [Pb^2+^] (M)	Critical [Pb^2+^] (g/100 mL)
Pb Phosphate	Pb_3_(PO_4_)_2_	8.0 × 10^−43^	whitish	4.48 × 10^−9^	9.28 × 10^−8^
Pb sulfide	PbS	3.0 × 10^−28^	silvery, metallic crystals or black powder		
Pb hydroxide	Pb (OH)_2_	1.43 × 10^−20^	white	1.53 × 10^−7^	3.17 × 10^−6^
Pb carbonate	PbCO_3_	7.4 × 10^−14^	colorless, rhombic crystals	2.72 × 10^−7^	5.64 × 10^−6^
Pb iodide	PbI_2_	8.49 × 10^−9^	yellowish crystalline solid		
Pb sulfate	PbSO_4_	1.6 × 10^−8^	white heavy crystal powder	1.26 × 10^−4^	2.61 × 10^−3^
Pb fluoride	PbF_2_	3.3 × 10^−8^	white to colorless crystals	9.38 × 10^−3^	1.94 × 10^−1^
Pb chloride	PbCl_2_	1.6 × 10^−5^	white solid or crystalline powder	1.59 × 10^−2^	3.29 × 10^−1^
Pb bromide	PbBr_2_	7.9 × 10^−5^	white, crystalline powder	2.70 × 10^−2^	5.59 × 10^−1^
**Pb Load: 3.77 × 10^−4^ (M)**					

Source: [28,32,52].

## Data Availability

The original contributions presented in this study are included in the article. Further inquiries can be directed to the corresponding author.

## Abbreviations

The following abbreviations were used in this manuscript:

iPb	Inorganic lead
Pb	Lead
DIW	Deionized water
BLL	Blood lead level
CDC	Centers for Disease Control
SSE	Sum of square
MSE	Mean square error
ANOVA	Analysis of variance
PPE	Personal Protective Equipment
ICP MS	Inductively Coupled Plasma Mass Spectroscopy
SD	Standard deviation

## Appendix A

**Table A1:** Constituents of formulated artificial sweat solution and their respective concentrations.

Constituents	Conc (mg/L)	Constituents	Conc (mg/L)
**Primary electrolytes and ionic constituents**		L-(+)-Glutamic acid	54.4
Sodium sulfate	58.3	Glycine	29.3
Sodium iodide	0.0106	L-Histidine	80.7
Sodium fluoride	0.462	L-Isoleucine	223
Sodium bromide	0.237	L-Leucine	27.5
Cadmium chloride anhydrous	0.0033	L-(+)-Lysine Monohydrochloride	27.4
Copper (II) chloride dihydrate	0.16	L-(+)-Ornithine Monohydrochloride	25.3
Iron sulfate heptahydrate	2.72	L-Phenylalanine	21.3
Manganese (II) chloride	0.138	L-Threonine	53.6
Sodium bicarbonate	252	L-(–)-Tryptophan	11.2
Potassium chloride	455	L-Tyrosine	30.8
Magnesium chloride hexahydrate	16.7	L-Valine	29.3
Sodium phosphate anhydrous monobasic	48.4	**Nitrogenous substances**	
Phosphorous pentachloride	2.71	Ammonium hydroxide—1 M solution	0.202
Sodium chloride	1550	Uric acid	9.92
**Organic acids and carbohydrates**		Urea	601
Lactic acid—1 M solution	1260	Creatinine	9.5
Pyruvic acid	15.9	Creatine monohydrate	2.24
Butyric acid	0.211	**Vitamins**	
Acetic acid	7.81	Thiamine hydrochloride	169
Hexanoic acid	0.105	Riboflavin	0.00489
Propionic acid	0.259	Nicotinic acid	5050
Isobutyric acid	0.0705	D-Pantothenic acid calcium salt	2480
Isovaleric acid	0.112	Pyridoxine hydrochloride	0.00206
D (+)-Glucose	30.6	Folic acid	0.00706
**Amino acids**		L-(+)-Ascorbic acid	17.6
DL-Alanine	51.1	Dehydroascorbic Acid	1.91
L- (+)-Arginine	136	Inositol	0.288
L- (+)-Aspartic acid	45.3	Choline chloride	3.63
L- (+)-Citrulline	70.1	P-Aminobenzoic acid	0.00973

## Appendix B

**Table A2:** Dissolution assay results for triplicate measurements of dissolved Pb in deionized water (temperature: 32.3 °C).

Time (mins)	% Dissolved Pb			Average	Std Dev	Average
	First	Second	Third			Conc (µg/mL)
0	0	0	0	0	0	0
5	73.4982	76.1874	81.1507	76.9454	3.8821	159.0098
15	87.8018	91.8177	94.8947	91.5047	3.5568	189.5655
30	93.8716	95.0891	95.3803	94.7804	0.8004	196.8371
60	97.8752	96.8316	95.4345	96.7138	1.2246	201.3535
90	98.2461	97.7863	96.1328	97.3884	1.1115	203.2699
120	99.6372	100.7308	98.2057	99.5246	1.2663	208.2534
180	100.7608	101.1573	99.5650	100.4944	0.8289	210.8181

**Table A3:** Dissolution assay results for triplicate measurements of Pb in artificial sweat (pH: 5.3, temperature: 32.3 °C).

Time (mins)	% Dissolved Pb			Mean	Std Dev	Average
	First	Second	Third			Conc (µg/mL)
0	0	0	0	0	0	0
5	48.9008	48.11603	46.04861	47.68847	1.4733	95.7279
15	75.94757	62.57971	61.16186	66.56305	8.1581	134.0323
30	73.66380	66.42962	64.68273	68.25872	4.7617	137.7511
60	71.96222	62.42384	65.60258	66.66288	4.8568	134.9069
90	70.45599	61.42785	64.24273	65.37552	4.6195	132.6323
120	68.82304	62.25351	65.44020	65.50559	3.2853	133.2191
180	66.42407	62.49837	67.07544	65.33263	2.4761	133.1947
